# Systematic characterization of wing mechanosensors that monitor airflow and wing deformations

**DOI:** 10.1016/j.isci.2022.104150

**Published:** 2022-03-22

**Authors:** Joseph Fabian, Igor Siwanowicz, Myriam Uhrhan, Masateru Maeda, Richard J. Bomphrey, Huai-Ti Lin

**Affiliations:** 1 Imperial College London, London, SW7 2AZ, UK; 2 The University of Adelaide, Adelaide, South Australia, 5005, Australia; 3 HHMI Janelia Research Campus, Ashburn, Virginia, 20147, USA; 4 Royal Veterinary College, Hatfield AL9 7TA, UK

**Keywords:** Entomology, Animal physiology, Sensory neuroscience, Biomechanics

## Abstract

Animal wings deform during flight in ways that can enhance lift, facilitate flight control, and mitigate damage. Monitoring the structural and aerodynamic state of the wing is challenging because deformations are passive, and the flow fields are unsteady; it requires distributed mechanosensors that respond to local airflow and strain on the wing. Without a complete map of the sensor arrays, it is impossible to model control strategies underpinned by them. Here, we present the first systematic characterization of mechanosensors on the dragonfly’s wings: morphology, distribution, and wiring. By combining a cross-species survey of sensor distribution with quantitative neuroanatomy and a high-fidelity finite element analysis, we show that the mechanosensors are well placed to perceive features of the wing dynamics relevant to flight. This work describes the wing sensory apparatus in its entirety and advances our understanding of the sensorimotor loop that facilitates exquisite flight control in animals with highly deformable wings.

## Highlights

• Dragonfly wings are innervated by an extensive collection of sensory neurons
• Mechanosensors are spread across the whole span of the wing with consistent patterns
• The axons of wing sensory neurons are scaled to compensate for transmission latencies
• Anatomically accurate models reveal wing strain fields that inform sensor distribution

## Graphical abstract

**Figure:**
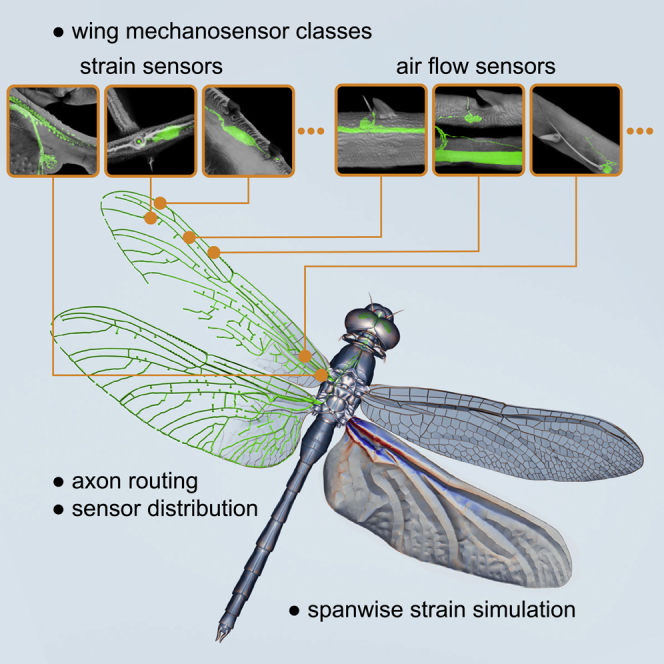

## Introduction

Compared to most engineered wings, wings in nature have a larger range of bending and twisting during flight. While some deformations are simply a consequence of light-weight construction, others are beneficial for flight. Passive wing twists and load-induced camber in locusts facilitate flight economy by preventing leading-edge airflow separation (Young et al., 2009). Similar aeroelastic deformations occur in the hummingbird’s wings for upstroke lift production (Warrick et al., 2005), and artificially stiffening bumblebee wings reduce their lift generation by 8.6% (Mountcastle and Combes, 2013). Dragonfly wings are exceptionally damage tolerant thanks to their elastic elements and network of crossveins (Rudolf et al., 2019). Moreover, some animals actively control their wing morphing via actuation and wing trajectories that enhance these functional benefits. Swifts continuously change their wing shape to reduce sink speed and maximize turning rates (Lentink et al., 2007). Bats actively control their wing deformation to improve aerodynamic performance (Swartz et al., 2007) (Riskin et al., 2008), which has led to biomimetic morphing wing applications (Wang et al., 2015) (Colorado et al., 2012). Because shape change, inertial forces, and aerodynamics loads are intrinsically linked, sensory feedback must be tailored to each wing accordingly. It is important to monitor buckling and/or flow fields with sufficient temporal and spatial resolution in order to provide fast and sufficiently comprehensive mechanosensory feedback to the flight controller. How do flying animals monitor their wing state in real time to maximize the benefits of deforming wings?

Vision is the best known sensory modality supporting the control of animal flight. Inertial sensing has also received attention, with a particular focus on rapid strain sensing in flies’ oscillating halteres and the vestibular system in flying vertebrates. However, there are further cues that are relatively understudied, yet likely crucial to flight control: sensing airflow and aeroelastic buckling of the wings themselves. The wings of flying animals experience combined inertial-aerodynamic (aeroelastic) loads, Coriolis forces, airflow stagnation and separation, and non-linear phenomena of vortex growth and shedding (Bomphrey and Godoy-Diana, 2018) (Combes and Daniel, 2003). Much of what we know about the mechanical feedback of wings in flight consists of isolated reports of specific sensor types on animal wings. The feather follicles of birds sense feather motion under aeroelastic forces (Brown and Fedde, 1993) (Carruthers et al., 2007), while the wings of bats and moths are populated with airflow and strain sensors which are known to contribute to body stabilization (Sterbing-D’Angelo et al., 2011) (Dickerson et al., 2014). Animal wings express a diverse array of mechanosensors each capturing a point measurement, yet a complete description of wing sensor arrangement on any highly deformable wing has been elusive. Consequently, any functional analysis on wing mechanics, interpretations of wing sensory signals, and modeling of feedback control strategies has been somewhat hypothetical. To understand the wing control strategy, we need to know what mechanosensors are found on the wing, how they are wired and distributed. Therefore, a systematic characterization of wing mechanosensors is needed for detailed investigations of the wing sensorimotor control loop.

Insect wings are driven by the musculature at the wing base, so all deformations on the wing are determined by the wing base cuticular configuration and dynamic states of the wing blades. Combining this minimal control input with a relatively simple nervous system, insects make a tractable model to study the sensorimotor control of deforming wings. Flapping flight relies on the phasic generation of precise aerodynamic forces through coordinated wing movements. Dragonflies are especially interesting due to their four independently controlled wings, each with adjustable amplitude, frequency, and angle of attack (Rüppell, 1989). This enables a large wing state-space, each with unique, time-dependent, aerodynamic, and inertial characteristics. Dragonflies can fly by synchronized flapping of all four wings, out-of-phase flapping of forewings and hindwings, flapping of the hindwings only, gliding, and even in mechanically linked tandem mating flights. Practically, dragonflies’ exposed wing veins and transparent, scaleless membranes facilitate the characterization of external and internal anatomy.

Sensory feedback from insect wings has been studied by neuroscientists and engineers since the early 1900s (Vogel, 1911) (Lehr, 1914) following the discovery of mechanosensors on butterfly wings. Further electrophysiological studies described afferent signals from mechanosensors called campaniform sensilla in fly wings (Dickinson, 1990). These neurons tend to fire a single action potential per wing stroke, so they cannot provide continuous strain estimation. Instead, strain is believed to be encoded predominantly through spike timing, which varies with the neurons spiking strain threshold, and the temporal dynamics of local strain (Dickinson, 1990) (Yarger and Fox, 2018). Flies’ hindwings have evolved into specialized club-like structures called halteres. The halteres express strain sensors which allow estimation of the insect’s body rotation through the detection of the Coriolis forces (Pringle, 1948). All flapping insect wings experience Coriolis forces, and recent work has demonstrated that strain sensor arrays on wings also function as inertial sensors (Pratt et al., 2017) (Jankauski et al., 2017). To detect body rotation through a flapping wing, only few strain sensors are needed (Hinson and Morgansen, 2015). This is consistent with the sparse distribution of campaniform sensilla found in some insects (Dickinson and Palka, 1987). However, wing-mounted sensors are also well positioned to monitor the instantaneous aeroelastic deformation and local flow velocities on the wings themselves. This line of investigation is far less developed yet critical for our understanding of flight control in flying insects and vertebrates, and serves as inspiration for novel engineered flight controllers.

To pave the way for comprehensive and mechanistic studies of animal wing sensing and control, we thoroughly examined Odonata (dragonfly and damselfy) wing sensors and answered the following questions: 1) How do sensory neurons innervate lightweight insect wings? 2) What types of sensors are found on an Odonatan wing? 3) How many sensors are on the wing and where are they located? 4) Are there any morphological adaptations to account for varying sensory latencies coming from different parts of the wing? 5) Are sensor distributions consistent across species of Odonata? 6) How does wing venation impact the mechanical strain distribution and therefore the strain sensory encoding? Taken together, this work systematically characterizes the wing mechanosensors, and enables future modeling of the sensorimotor loop for exquisite wing control.

## Results

### Sensory neurons inside Odonata wings

#### Which regions of the wing blade are innervated by sensory neurons?

**Figure 1:**
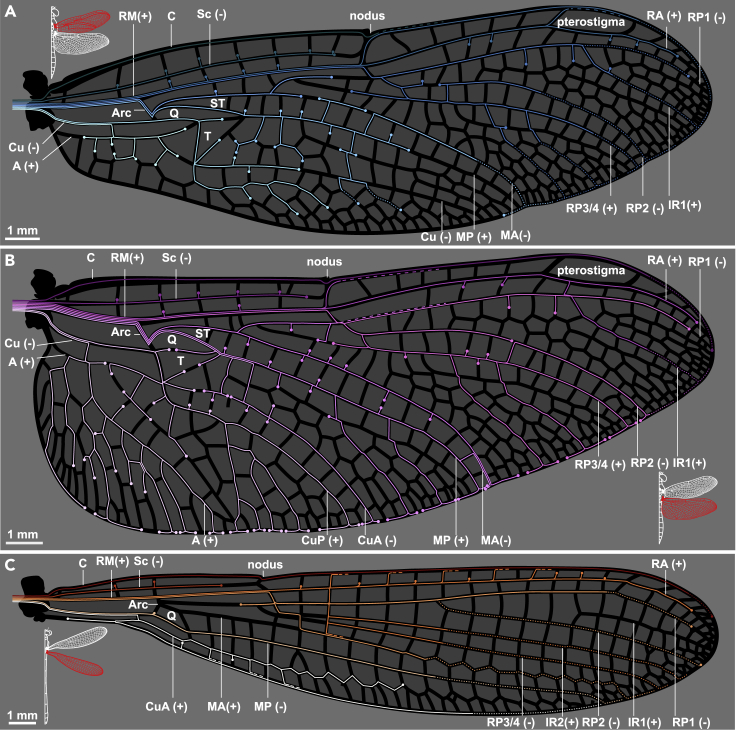
The axon routing pattern of selected odonates Neuronal innervation diagram of male eastern amberwing dragonfly (*Perithemis tenera*) forewing (A), hindwing (B), and a male blue-fronted dancer damselfly (*Argia apicalis*) hindwing (C). A dedicated afference wing nerve branches out into different veins from the wing base. The color tones of axonal tracks were chosen arbitrarily for readability. They do not imply hierarchy or relationship of the tracks. Dots indicate terminal mechanosensory cell body for each track; dashed lines denote merging tracks; dotted lines represent tracks that were interpolated from incomplete back-fill images and mechanosensors found on the veins. Major veins and structural elements of the wings are labeled: C- costa; Sc - subcosta; RM - radius/media; Cu - cubitus; (A) anal; Arc - arculus; T - triangle; ST - supratriangle; Q - quadrilateral. The corrugations of the wings/topology of veins are represented by “+” for “ridges” (dorsally convex) and “-” for “valleys” (dorsally concave). See also Figure S1.

To visualize the neuronal innervation within Odonata wings, we imaged the complete collection of wing neurons and associated cuticular structures via confocal microscopy (see Methods). For comparison, we visualized wings from a small dragonfly species *Perithemis tenera* and a similar-sized damselfly *Argia apicalis*. Some of these neurons’ axons run the entire wing length, making them among the longest in insects. All axons follow a relatively direct path and terminate with a soma which is often directly under an external cuticular structure (Figures 1A and 1B). Unlike the leading edge and the longitudinal veins, the trailing edge of *P. tenera* wings has no dedicated axon tract. Instead, it is innervated locally by extensions of the closest longitudinal vein tracts. The venation of damselfly wings consists of well-aligned rectangular cells, resembling a modern city grid. The innervation pattern of the damselfly (*A. apicalis*) wing is similar to that of the dragonfly (*P. tenera*), with some exceptions (Figure 1C). Unlike the dragonfly wing, the trailing edge of the damselfly wing is innervated directly, with a tract of axons running along the length of the trailing edge.

### Variation and optimization of axon routing in the wing

#### How stereotyped is the axonal routing and what type of variations exist?

**Figure 2:**
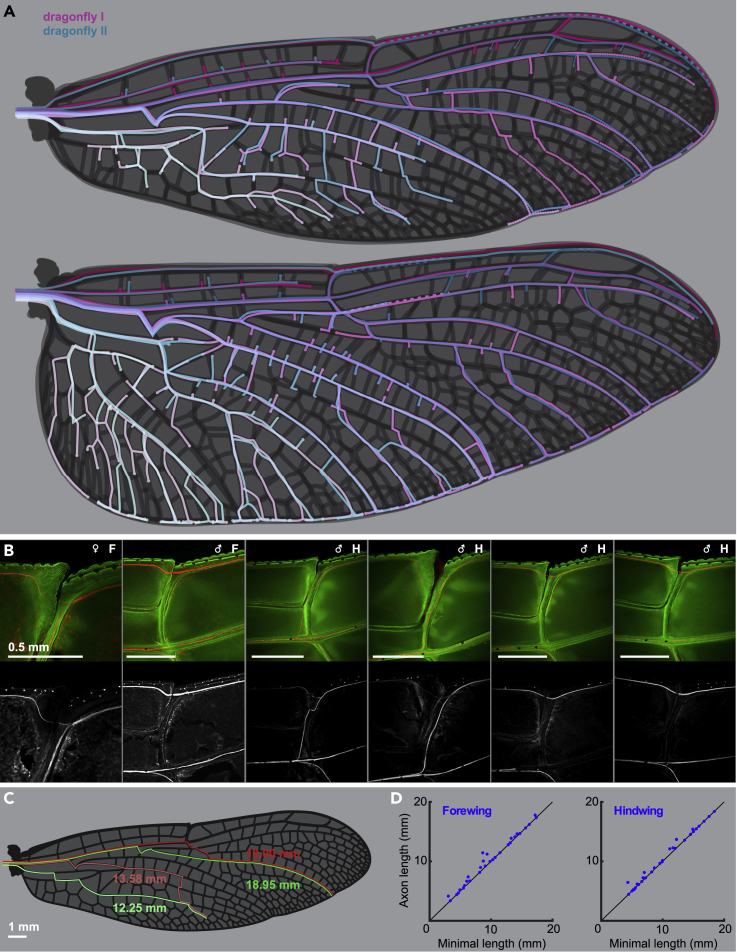
Variations in the axon routing patterns of dragonfly wings (A) Variability of axonal tracks in two dragonflies (*Perithemis tenera*) fore- and hindwings. The dragonfly II wings were superimposed on the dragonfly I’s, scaled, and warped in Adobe illustrator to align the wing margins and main veins. Each vein carries a single nerve. (B) Variability of the axonal track passage at the nodus of *P. tenera*: axons of the leading-edge wing margin sensors distal to nodus may continue down the costa or take an alternative path down the radius vein. The path does not seem to depend on the sex of the insect and the variability occurs in both fore (F) and hind (H) wings. (C) Real paths (red) and minimal distance paths (green) of axons innervating a wing margin bristle-bump complex neuron (anterior pair) and a campaniform sensillum (posterior pair) in a male (dragonfly I) forewing. (D) The real path distance and minimal path distance for 31 pseudorandomly selected sensors on the forewing and hindwing.

The complex venation pattern of dragonfly wings allows many different axon routing options. We compared the routes axons take in a set of male and female *P. tenera* wings and highlighted regions where we observed differences in axon routing (Figure 2A). We performed a larger survey of routing variation at a key structure of Odonata wings: the unidirectional flexion joint (i.e. nodus). The nodus contains a significant amount of elastic resilin, a material which allows larger flexion and elastic energy storage during wing motion (Rajabi et al., 2017a) (Mamat-Noorhidayah et al., 2018). Passing an axon bundle across an articulating joint is more structurally complex than a fixed vein. Interestingly, we see that in some individuals, axon bundles from the proximal costa pass through the nodus joint, whereas in other individuals the distal costa is innervated via subcosta bundles, bypassing the nodus (Figure 2B).

We hypothesize that axon length minimization is a primary factor determining the organization of axon paths in the dragonfly wing. To test this, we pseudorandomly selected 31 evenly distributed sensors on the forewing and hindwing of one *P. tenera* sample and compared the true axon length for the selected path against the shortest feasible path from sensor to wing hinge (Figures 2C and 2D). Combining data from both wings, the total axon length to innervate the 62 selected sensors was only 3.3% longer than the shortest possible routing solution, with 53.2% of the axons taking the shortest viable path. Neurons which took suboptimal routes for axon length minimization tended to make common “mistakes”, particularly around the arculus and nodus, regions that play important structural roles in flapping flight (Wootton, 1992). Our data suggest that the organization of axons in the dragonfly wing is well optimized to reduce total wiring length, within the constraints of the mechanical roles of different wing veins.

### Wing sensor morphologies and classification

#### What sensors are found on an Odonata wing?

**Figure 3:**
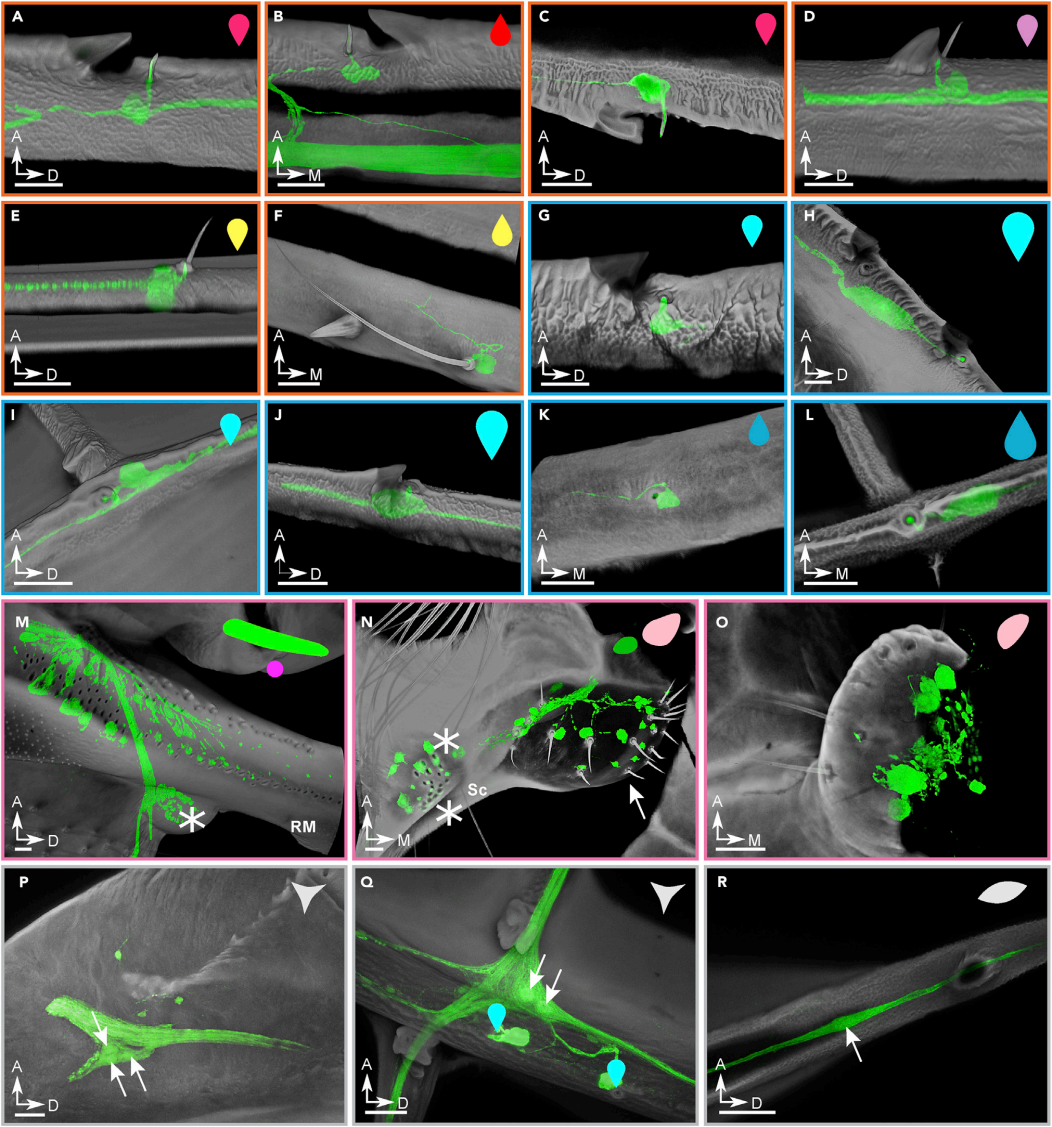
The classification and morphology of all wing sensors Examples of sensors found on the wings of the dragonfly *Perithemis tenera*. The scale bars are all 25 μm. (A-F) Candidate airflow sensors: (A) (B) (C) costa dorsal, costa ventral, and trailing-edge margin bristle complex. These three special examples show the double-innervated bristle which consists <25% of all the wing margin bristles. All other bristles have one dendrite innervating the bristle base (not shown). (D) Radius bristle-bump; all bristles of this type are innervated by one neuron at the base. (E) Short bristle of the type present on several major veins. (F) Long bristle of the type present on the medial part of major veins. (G–L) Strain sensors: (G) dorsal costa campaniform sensillum (CS); (H) large dorsal costa CS; (I) dorsal cross vein CS; (J) large radius anterior CS immediately distal to pterostigma; (K) ventral subcosta CS; (L) terminal ventral CS of the radius posterior 1 (RP1) vein. (M–O) Wing base sensory fields: (M) crevice organ; two parallel fields of directionally tuned elliptical CS at the base of radius/media (RM) vein. Asterisk marks the dorsal insertion site of a wing base chordotonal organ; (N) hair plate (arrow) and two adjacent CS fields (asterisks) ventrally at the base of subcosta; (O) hair plate ventrally at the base of cubitus. (P–R) Multipolar and bipolar receptors: (P) multipolar receptor at the base of costa; arrows point to/indicate cell bodies; (Q) multipolar receptor located at the junction of the anal vein and the first/medial cross vein connecting it to cubitus. Two adjacent dorsal CS are indicated with cyan markers. (R) Bipolar receptor. All example images are from the right forewing, with cuticle in gray, and neurons in green. The symbols in the upper right corner of each panel are notations to show sensor type and dorsal/ventral placement which will be used in Figuers 4 and 5. See also Figure S2.

Odonata wing membranes are smooth, but their veins are covered in microscopic structures with different roles (Gao et al., 2013). To identify sensory structures, we combine imaging data of external morphologies and internal anatomy. Based on our current understanding of insect wing sensors, we classified the wing sensory structures into eight classes: wing margin bristle complexes (Figures 3A–3C), bristle-bump complexes (Figure 3D), isolated bristles of varying length (Figures 3E and 3F), campaniform sensilla with associated structures (Figures 3G, 3H, and 3J), isolated campaniform sensilla (Figures 3I, 3K, and 3L), campaniform sensilla fields (Figures 3M and 3N), hair plates (Figures 3N and 3O), and multipolar cells (Figures 3P–3R).

The costa vein (leading edge) and trailing edge of the wing are densely populated with sensory bristles (∼30 μm long in *P. tenera*), associated with dorsal or ventral serrations (Figures 3A–3C). Similar bristles have been found on the wing margin of moths, sensitive to directional vibratory airflow (Yoshida and Emoto, 2011), and in Drosophila, where stimulation triggers a defensive leg kicking response (Li et al., 2016). In the dragonfly, each bristle attaches to the dendrite of a sensory neuron at its base, and in up to 25% of cases, a second sensory neuron sends its dendrite through the shaft of the bristle to its tip. These are the only external wing sensors that are innervated by more than one neuron in Odonata.

The thickest longitudinal veins (subcosta, radius, cubitus, and media) contain short (10–15 μm) bristles, each paired with a small saw-tooth-shaped bump (Figure 3D). The shape of these paired bumps is highly stereotyped across the wing, and across multiple species, as is the spatial relationship between the bump and bristle. Each bristle lies several micrometres distal from the bump, which protrudes from the vein at a shallow gradient on its medial side, and a steep gradient on its distal side.

**Figure 4:**
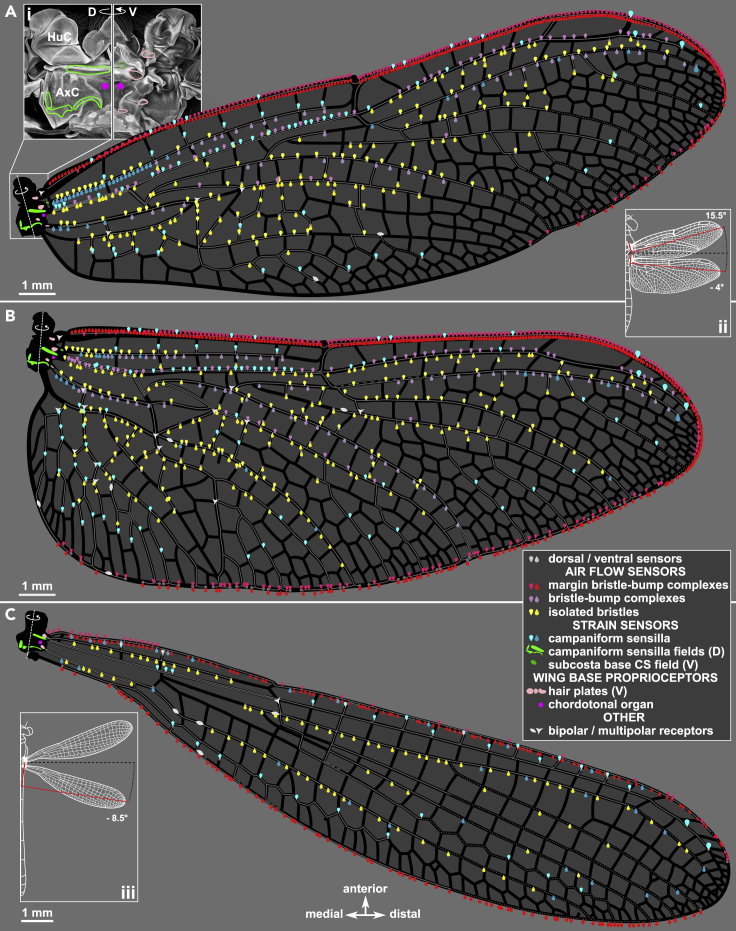
A sensory map of odonate wings Distribution of all confirmed sensors on the *Perithemis tenera* dragonfly fore- (A) and hind (B) wings and a hindwing of *Argia apicalis* damselfly (C). All sensor notations follow Figure 3 and the figure legend. Inset (i): maximum intensity projection showing *P. tenera* right forewing base dorsally and ventrally with the sensor fields outlined. Insets (ii) and (iii): diagrams showing the wings’ natural resting sweep angles; the red reference lines mark the wing span axis perpendicular to the anatomical wing hinges.

A variety of strain-sensitive campaniform sensilla are found on the costa (Figures 3G and 3H), and several other longitudinal veins (Figures 3I–3L), often paired with the same type of saw-toothed cuticular bumps described for short bristles. We observe isolated campaniform sensilla on many of the joints connecting the longitudinal radius vein to cross veins (Figure 3L). At the base of the radius, there are five fields of campaniform sensilla (Figure 4A), with each sensillum innervated by an associated sensory neuron (Figure 3M). These sensor fields match the crevice organs described by Simmons (Simmons, 1978), sensors whose name at the time reflected their resemblance of small pits on the cuticle. Fields of campaniforms with high aspect ratio pits are associated with directional strain sensing (33, 34). Specifically, two such fields on the subcosta have an almost orthogonal directional bias which is conserved across odonates. We also identify several hair plates on the wing base (Figures 3N and 3O), as well as a chordotonal organ, identified by its characteristic anatomy (35, 36) (Figure 3M). These sensory structures are typically associated with proprioception in insects (37). Finally, we observe multiple multipolar neurons located at some joints between wing veins (Figures 3Q and 3R) with unknown function and no associated cuticular structure. For more examples, please see Figure S2.

### A complete sensor survey of Odonata wings

#### Exactly how many sensors are on the wing?

Excluding the wing base campaniform fields, we found a total of 771 sensors on the forewing and 894 sensors on the hindwing of a dragonfly (*Perithemis tenera*), and 358 sensors on the hindwing of a damselfly (*Argia apicalis*) (Figure 4). Extrapolating from this, we estimate this small dragonfly has well over 3000 wing sensors on its four wings, and the damselfly approximately half as many. We found a total of 84 and 98 isolated campaniform sensilla on fore- and hindwing of *Perithemis tenera*, respectively, with a bias toward the dorsal surface (Table S1). The abundance of each sensor type varies considerably, and bristles significantly outweigh campaniform sensilla on most veins. Interestingly, the damselfly wings studied are completely devoid of bristle-bump complexes or dorsal bristles (Figure 7C).

### Neuron size and axon length

#### Is there any morphological adaptation to account for the sensory latency of wing sensory neurons?

**Figure 5:**
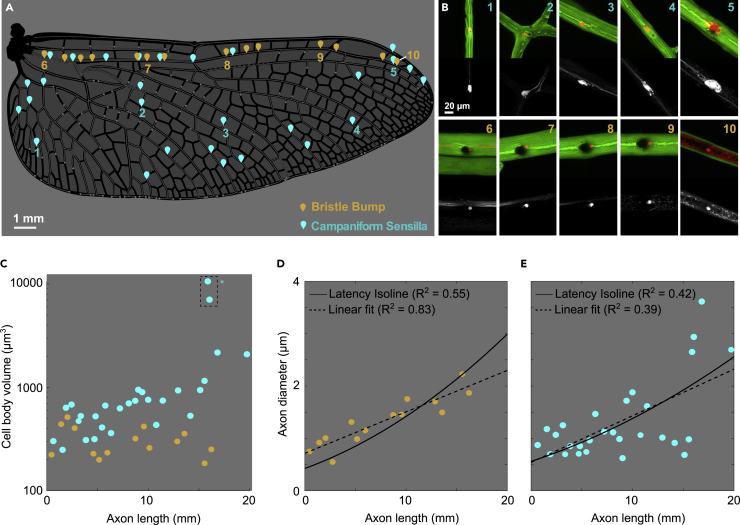
Quantifying the cell body volume and axon diameter of wing neurons (A) Positions of wing sensors (selected to evenly sample the wingspan) on the right hindwing of *Perithemis tenera*. Cyan—campaniform sensilla (CS) orange—bristle-bump complexes. (B) Example images of the cell body and axon of wing sensors, with cuticle in green and neurons in red. Grayscale images underneath show the neuron channel alone. (C) Cell body volume for wing sensors outlined in (A). (D and E**)** Axon diameter for bristle-bumps (D) and campaniform sensilla (E), black line indicates the axon diameter required to achieve equal response latency for different axon lengths, dashed line indicates simple linear fit.

The dragonfly wing is a long structure, and the axon lengths between sensors on proximal or distal regions of the wing (Figures 5A and 5B) could introduce significant temporal differences in spike arrival at the thoracic ganglia. While such difference can be accounted for in the sensory representation scheme, a better solution is to remove the temporal difference entirely through anatomical modifications. Specifically, distal sensors can compensate for their longer axons by increasing conduction speed via enlarged axon diameters. We measured cell body volume and the axon diameter for sensors at different spanwise locations on the wing. Cell body volume is approximately constant for bristle-bumps, but tends to scale with axon length for campaniforms (Figure 5C), with two abnormally large cells located near the wing tip, which we refer to as “giant wing tip campaniforms”, highlighted by a dashed box. We found that axon diameters increase with axon length, both for bristle-bumps (Figure 5C) and campaniform sensilla (Figure 5D). Based on the cable theory for neural conduction speed (Kandel et al., 2000), we derived a relationship between the axon diameter and length for “isochronic” scaling (see Methods). The axon diameter of bristle-bumps and campaniform sensilla loosely follows our latency isoline (R^2^ = 0.55 and R^2^ = 0.42, respectively), as well as a simple linear fit (R^2^ = 0.83 and R^2^ = 0.39) suggesting some level of latency compensation in these mechanosensors.

### Giant campaniform sensilla near the pterostigma

#### Are wing tip campaniform sensilla universal across odonates?

**Figure 6:**
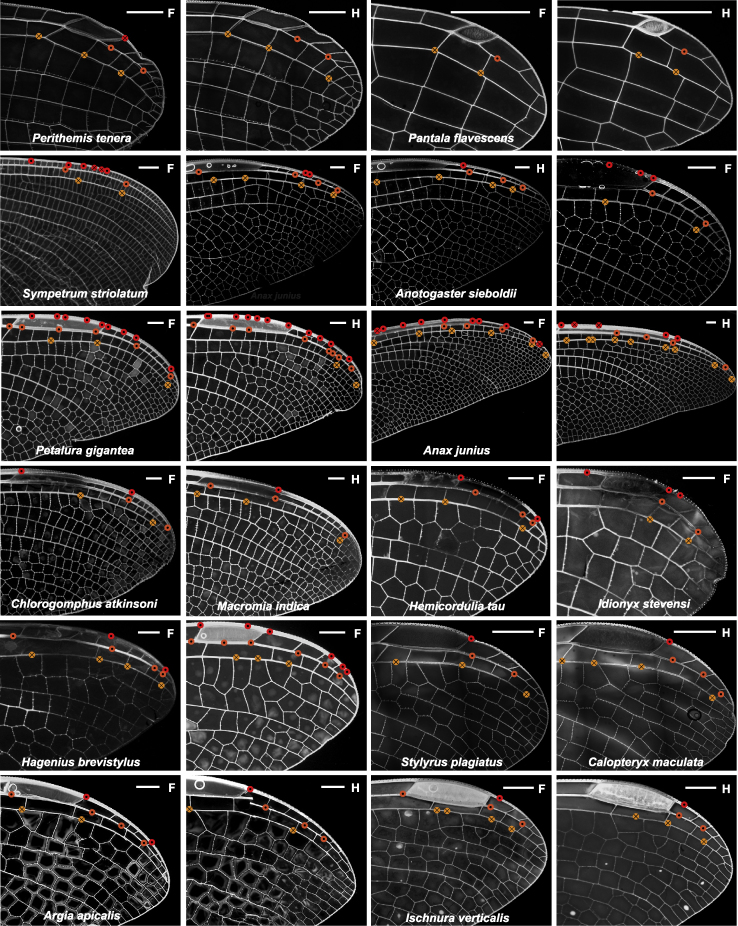
Positions of campaniform sensilla at the wing tips of studied odonates Campaniforms on costa (red), radius anterior (dark orange), and radius posterior (light orange) are marked. Dots denote dorsal, crosses – ventral sensors. Enlarged markers indicate campaniform sensilla innervated by sensory neurons possessing significantly larger soma as seen in backfills for *Argia apicalis* and *Perithemis tenera*, and inferred from a prominent bulge on the vein in *Ischnura verticalis*, likely expanded to accommodate a large cell body. Scale bars: 1 mm.

We systematically searched for campaniform distribution patterns near the wing tip across 15 Odonata species (Figure 6). All but one dragonfly species studied (*Anax junius*) have an isolated campaniform sensillum immediately distal to the pterostigma on the costa and radius veins, but they were absent in all three damselfly species. In *P. tenera* where we performed internal neuron size measurements, these two giant campaniform sensilla can be seen in Figure 5. The cell bodies are so large that we observe a significant bulge in radius vein to accommodate them (Figure S2F).

### Sensor distribution across Odonata species

#### Do sensor densities inform consistent patterns across odonates?

Odonata is a diverse insect order, phylogenetically close to the first flying insects, with wingspans, wingbeat frequencies, and flight speeds varying significantly across species (Bomphrey et al., 2016). How consistent are the classes and distribution of wing mechanosensors across different species of Odonata? We imaged the external morphology of wing sensors in 15 species (12 dragonflies and three damselflies) from 10 of the most studied families of odonates. Our analyses focused on three major longitudinal veins: the costa, subcosta, and radius, as they are anatomically similar across all species, express a diverse range of sensors, cover most or all of the wingspan, and bear most of the load during flight (Büsse and Hörnschemeyer, 2013) (Wootton, 1992).

**Figure 7:**
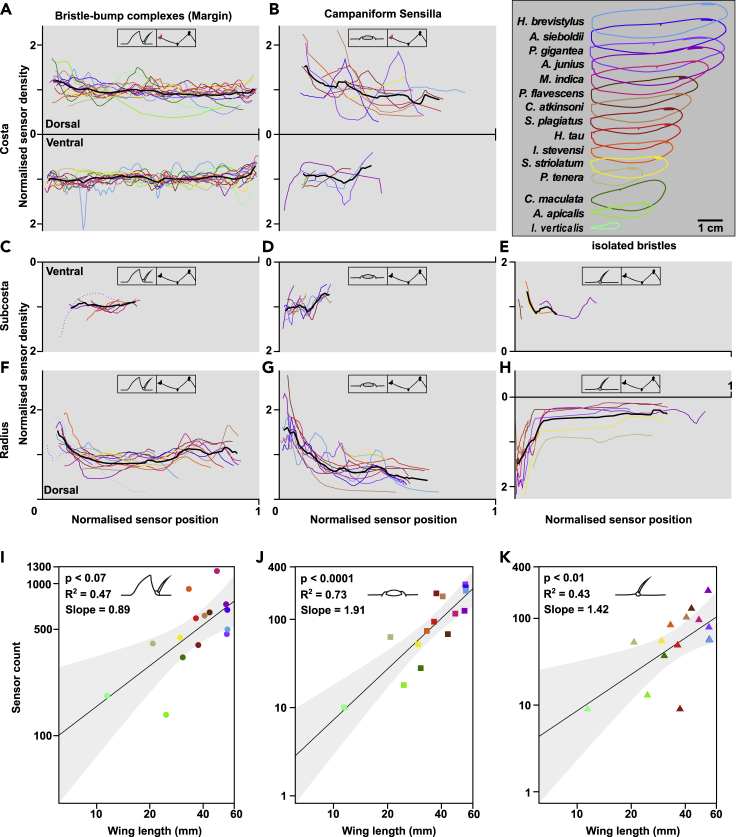
A comparison of selected sensor distribution across odonate families Sensor density distribution (A–H) and sensor count (I–K) across 15 odonate species. Normalized sensor density is shown for dorsal and ventral side over normalized sensor position on three longitudinal veins, costa (A and B), subcosta (C–E), and radius (F–H) (See Table S1 for sensor count values). The insets in each panel highlight sensor type and the respective vein. Sensor density is normalized by the mean of each density curve. Sensor position is normalized by the length of the respective right forewing. The black bold line shows the mean of all species in each plot. (I–K) Sensor count is the sum for each sensor type of all three longitudinal veins. The sensor count is plotted over the respective wing length for each species. Black line shows the ordinary least squares (OLS) regression and the gray shaded area shows the 95% confidence interval. Each data point is color coded by the respective species (see legend in upper right panel).

All species studied maintain the same relationship between wing corrugation and sensor class (Figures 7A–7H). Bristle-bump complexes and campaniform sensilla are found on the ridges (ventral side of subcosta and dorsal side of radius) and isolated bristles in the valleys (dorsal side subcosta and ventral side radius) (Figures 4A and 4B). Margin bristle complexes and wing base campaniform fields are found in all 15 species (Figure 7A), but bristle-bump complexes (Figures 7C and 7F), campaniform sensilla (Figures 7B, 7D, and 7G), and isolated bristles (Figures 7E and 7H) are missing in some species. The differences across species are most evident on the subcosta, where only dragonfly species express bristle-bump complexes and isolated bristles (Figures 7C and 7E).

On the costa, the normalized density of wing margin bristles is relatively constant across the wing length for all species (Figure 7A). However, the damselflies *Ischnura verticalis* and *Calopteryx maculata* and the dragonflies *P. tenera*, *Stylyrus plagiatus* and *Idionyx stevensi* show local density peaks near the nodus (approximately half wing length) on the dorsal side. For all species but *Petalura gigantea*, sensor densities are slightly higher on the ventral side compared to the dorsal (Table S1). Smaller wings have higher wing margin bristle-bump sensor density, with *I. verticalis*, *P. tenera*, and *I. stevensi* having an average density of 7 sensors/mm, 8 sensors/mm, and 10 sensors/mm, respectively, compared to 4 sensors/mm average density for *A. sieboldii* and *Hagenius brevistylus* on the dorsal side of the costa. The density of campaniform sensilla along the major veins decreases gradually from wing hinge to tip (Figures 7B, 7D, and 7G).

The cross-species comparison reveals that sensor counts increase with wing length for all three sensor types (Figures 7I–7K). Given the data available, the campaniform sensilla counts scale faster than the isometric line (OLS slope 1.91; 95% CI [1.18, 2.6]), and has the tightest correlation compared to bristle-bump complex (OLS slope 0.89; 95% CI [0.29, 1.5] and isolated bristle (OLS slope 1.42; 95% CI [0.39, 2.45]).

### The strain fields and venation patterns

#### How does the wing venation impact mechanical strain distribution and therefore the encoding of strain?

Insect wings are lightweight but sufficiently stiff to support aerodynamic and inertial loads. Their intricate venation and extremely thin membrane (∼10 μm thickness) make them challenging to image and model. As a result, insect wings are often simplified to thin plates with uniform thickness for aerodynamic or structural modeling (Liu et al., 2016). In reality, strain fields on the wing do not spread uniformly. Instead, they are channeled through the veins which also happen to house all the mechanosensors. To visualize how the venation pattern impacts strain fields during flapping, we created the most anatomically accurate dragonfly wing model to date for deformation analyses.

**Figure 8:**
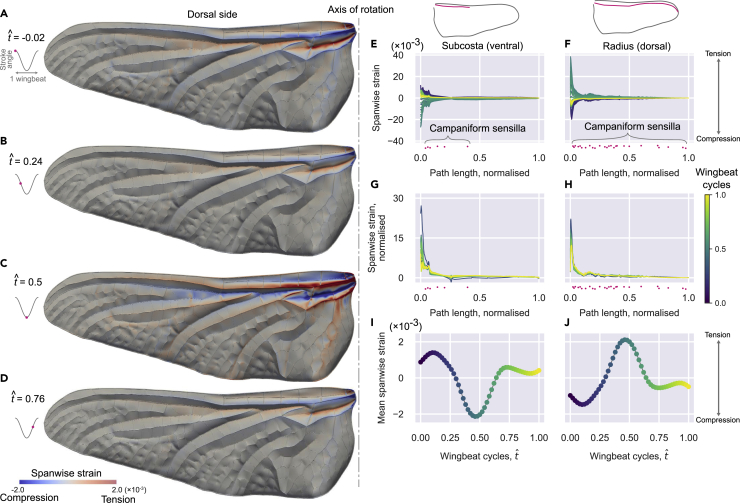
Strain field propogation on a simulated flapping wing (A–D) The hindwing of a *Sympetrum striolatum* was reconstructed in 3D for this structural analysis. Four time instances of the flapping cycle are presented: beginning of downstroke (A), mid-downstroke (B), beginning of upstroke (C), and mid-upstroke (D). A hindwing of a *Sympetrum striolatum* was reconstructed in 3D for this simulation. (E and F) Spanwise strain along ventral subcosta and dorsal radius where 962 campaniform sensilla can be found. The color map represents different phase of a flapping cycle. (G and H) Spanwise strains along the veins, each line is normalized by the mean of the strain along the vein at that time. The x axis (path length, or position along vein) for the spanwise strains are normalized with the total path length for the corresponding vein. (I and J) Temporal variation of the mean spanwise strain on the ventral subcosta and dorsal radius. The measured positions of campaniform sensilla are shown by magenta dots (E–H**)**. The vertical variation of the dots is purely for readability. See also Figure S3.

We constructed a finite element wing model from μCT data (see Methods) for a *Sympetrum striolatum* dragonfly hindwing. To understand the strain field propagation given the venation pattern, we subjected our model wing to a sinusoidal flapping oscillation around a single axis (parallel to the wing plane and perpendicular to the spanwise axis) in a vacuum. The surface spanwise strain contours of four snapshots (Figures 8A–8D) show a high concentration of strain at the longitudinal veins, especially near the wing base. The spanwise strain is greatest at the stroke reversals (Figures 8A and 8C); the proximal portion of the radius experiences compression (negative strain, in blue) at the transition from upstroke to downstroke (Figure 8A), while the same vein experiences tension (positive strain, in red) at the transition from downstroke to upstroke (Figure 8C). On the other hand, the proximal subcosta and cubitus (two longitudinal veins adjacent to the radius) have the opposite sign. This is because the corrugation offsets some longitudinal veins away from the neutral plane of bending. We also quantified the spanwise strain along two major longitudinal veins (subcosta and radius, Figures 8E–8J) where we found campaniform sensilla on their corresponding “ridge” sides (ventral side for subcostal and dorsal side for radius). Each of these veins shows a consistent spatial distribution of strain along their length (Figures 8G and 8H), but with a large temporal variation in magnitude over the flapping cycle (Figures 8I and 8J; Video S2).

## Discussion

We have presented the most comprehensive description of the sensory apparatus on the wings of a flying insect (Video S1). The mechanical state of a “rigid” wing (as an aircraft) can be described by its angle of attack, airspeed, and the configuration of its control surfaces. However, as the dragonfly wing twists in response to loads, the angle of attack varies along the wingspan with time and the local airspeed is not constant. Thus, to encode the state of a biological wing, detailed monitoring of both strain and flow sensing is required. Our findings answer numerous key questions on how mechanosensors in animal wings are organized to fulfill this task.

Video S1. Wing innervation and sensor distribution of an eastern amberwing dragonfly, *Perithemis tenera*, related to Figures 1, 2, 3, and 4 Wing innervation and sensor distribution of an eastern amberwing dragonfly, Perithemis tenera. Labelled neurons in the whole mount preparation of the wing imaged with a 5x objective are shown in green. Placement of five classes of wing sensors are shown as examples (teardrop markers): campaniform sensilla, wing margin bristle-bump complexes, bristle-bump complexes, isolated bristles and multipolar receptors. Representative sensors are shown in three-dimensional rotating views, generated from high-magnification confocal data.

### Metabolic investment in the wing sensory system

One of the largest contributions to the metabolic costs of neuronal signaling is the length of an axon (Niven and Laughlin, 2008) (Sterling and Laughlin, 2015). Longer axons increase a neuron’s volume, thus the electrolyte movement required to propagate an impulse. Maintaining this ionic balance makes up as much as 50% of the brain’s total energy (Grenell, 1958), so there are strong selective pressures for minimizing axon length. Another key contributor for metabolic costs is the total neuron count. Each wing sensor has a cost in terms of mass, moment of inertia, metabolic energy, and increased complexity for decoding. For this reason, we expect the sparsest sensor arrangements that provide sufficient performance and adequate robustness. Simulations of sparse strain sensor systems show that an optimized sensor array results in a significant reduction in sensor count (Mohren et al., 2018). Mechanical stress on wings is largest close to the wing base, and the metabolic cost of neuronal signaling increases in proportion to the length of an axon; therefore, a bias toward medial sensor placement could increase sensory system efficiency.

Dragonfly wing contains a relatively large number of sensory neurons with long axons. Even though our full-wing mapping data were collected from one of the smallest dragonfly species, sensor counts are higher than equivalents reported on the wings of moths, flies, or beetles described so far (Dickerson et al., 2014) (Cole and Palka, 1982) (Frantsevich et al., 2014). Despite the large sensor counts, strain sensors on the dragonfly wings still form sparser distributions posteriorly and distally. This shows that efficient sensor placement is still important, and odonates simply have a high level of metabolic investment in the wing sensory system.

### Flow sensing on the wing

Both the leading and trailing edges of Odonata wings are densely populated with sensory bristles, accounting for approximately 60% of all sensors on the wing. During flapping flight, dragonflies generate a strong leading-edge vortex which is shed during the downstroke (Bomphrey et al., 2016) and the wing edges tend to experience large fluctuating pressure gradients (Shumway et al., 2020) with cyclic changes of airflow direction. The bristles on the wing margin may, therefore, play a role in detecting the timing and intensity of vortex formation and shedding. Similar sensors exist in moths and are known to be sensitive to directional vibratory airflow (Ai et al., 2010).

(Houot et al., 2017)(Fabian et al., 2020)(Piersanti et al., 2014)(Yoshida et al., 2001) On radius vein, the bristle-bump sensory complex is a novel structure. The bump itself is not innervated, but it could condition the external flow stimulus acting on the bristle (unpublished data). The bump might also protect the small bristles, preventing damage from collisions or parasites. We see evidence that bristle-bumps exhibit isochronicity: the axon diameter varies with axon length, normalizing the conduction time of impulses so that all sensors report disturbances to the ganglia with similar latencies. Isochronicity has been reported in motor systems where co-contraction of all motor units is required (Seidl, 2014) (Lorenzo et al., 1990). This approach has also been observed in retinal ganglion cells and crayfish antennule (Stanford, 1987) (Mellon and Christison-Lagay, 2008). In the example of retinal ganglion cells, the computation of motion vision requires accurate spatiotemporal representation of neighboring receptive fields. The bristle-bump complex therefore may be monitoring precise spatiotemporal variations in air velocity on the wing surface.

Flow sensor types are clearly associated with local wing topography (e.g. corrugation); for example, long trichoid sensilla are almost exclusively found in the wing’s local valleys, while short bristles and bristle bumps lie on ridges. This arrangement is suitable to detect the eddies within the corrugations and the attachment state of airflow near the boundary layer (Bomphrey et al., 2016), respectively. Isolated bristles dominate the central part of the wing blade in both the dragonfly and the damselfly. The length of these bristles varies ten-fold, with bristle length decreasing from the wing base to the wing tip. Because the air speed increases from wing root to wing tip during flapping flight, this length distribution may match to the natural patterns of boundary layer height. However, it is important to note that flow fields around flapping wings are highly unsteady and may only intermittently follow this pattern. A more detailed aerodynamic study considering the distribution and morphology of the bristles is necessary.

Could sensory bristles on the dragonfly’s wings serve other purposes? We suspect the doubly innervated bristles at the leading edge (Figures 3A and 3B) might additionally serve as a chemosensor (Houot et al., 2017). Dragonflies are often considered anosmic due to their minuscule antennal lobes (Fabian et al., 2020), but there is behavioral evidence linking odors and predation site preference (Piersanti et al., 2014). Dragonflies are not able to groom their wings like a fly and their wings do not come into contact at rest, thus a tactile function is unlikely. Bristles are absent in the proximal 2/3rds of the forewing trailing edge which often come into contact with the hindwing during a flapping cycle. This pattern has been observed in the wing margin bristles of a butterfly’s forewing and hindwing (Yoshida et al., 2001), suggesting a common solution to avoid unwanted signals or damage caused by wing collisions.

### Wing strain sensory encoding

The isolated campaniform sensilla are sparsely distributed around the edge of the wing. This ring-like arrangement is consistent with capturing the twist of the wing blade efficiently, as the largest deformations occur at a distance from the axis of twist (48, 60). Sporadic campaniform sensilla are found along the leading edge, yet the most posterior campaniform sensilla are located some distance away from the actual trailing edge. This spacing may prevent sensor damage from wing wear (Hamed Rajabi et al., 2017b) and avoid noise from aeroelastic flutter. Campaniform sensilla counts on the wing of the grasshopper *Melanoplus sanguinipes* (sensilla count: 54 (Albert et al., 1976)) and the fruit fly *Drosophila melanogaster* (sensilla count: 62 (Cole and Palka, 1982) (Dickinson and Palka, 1987)) are both higher than the OLS regression prediction based on the dragonfly wings. This suggests that large wings are not simply a scaled-up version of smaller wings. The number of sensors and their specific spacing likely depend on the biomechanics and phylogenetic constraints.

The radius and axillary complex at the wing base are adorned with five fields of campaniform sensilla with 30–90 sensors per cluster (Figure 4A, inset). Both structures are load-bearing, transmitting forces from thoracic muscles to the wings and flight forces from the wings to the body. Severing either structure led to catastrophic failure of wing function (unpublished lab observations). Their sensilla have high aspect ratios (elongated oval shape instead of circular, unpublished data), indicating high directional selectivity (Delcomyn, 1991). Sensor orientations vary within each field, which might reflect loading patterns in the wing base during flapping and gliding flight. Similar fields of campaniform sensilla are found on both the wings and the halteres of flies (Cole and Palka, 1982). These sensors might contribute to inertial sensing and/or monitoring of wing loading.

How might the wing sensory system encode deformation? Given the structure and material properties of a wing, the strain distribution along the major veins is determined by the sum of inertial and aerodynamic loads. During flapping, inertial loads generally exceed the aerodynamic loads for large insects (Jankauski et al., 2017). To encode any aerodynamic components of strain, the wing sensory system must detect small variations of the strain field from the dominant baseline signal due to inertial loads. As the first step toward understanding this, we performed structural analysis on the most anatomically accurate dragonfly wing to date. The inertial loading shows strain field propagation along the wing veins with highly stereotyped spatial profiles (Figures 8G and 8H; Video S2). This baseline signal is almost constant in shape (as prescribed by the wing structure and kinematics) but varies in magnitude over time (temporal profile shown in Figures 8I and 8J). Any deviation from this profile suggests disturbances due to either aerodynamic events or motor modulation (e.g. manoeuvring).

Vidoe S2. Visualization of the spanwise strain for a common darter dragonfly, *Sympetrum striolatum*, in the flapping-wing structural dynamics simulation, related to Figure 8 Visualization of the spanwise strain for a common darter dragonfly, Sympetrum striolatum in the flapping-wing structural dynamics simulation. Spanwise strain surface contours in the wing coordinates (left) and spanwise strains on two major longitudinal veins (right). See Methods for the definitions. The wingbeat cycles shown are 21.75 ≤ t/T ≤ 30.75, starting from pronation.

To detect such variation requires high spatial resolution and dynamic range in strain sensitivity, which can only be accomplished by increasing the campaniform count. Specifically, because campaniform sensilla function as strain detectors, there are two approaches to encode high-resolution strain values. Firstly, clusters of campaniforms at key locations with different strain thresholds and temporal tuning can, collectively, cover a range of strain magnitudes. Alternatively, a population of similar campaniforms distributed along the vein can encode the magnitude of variation in their activation phase as the deformation propagates. This is more analogous to a visual system with relatively homogenous photoreceptors which detects motion via phase correlation. Both approaches require a high campaniform count for high resolution and are not mutually exclusive. While the observed distribution of campaniform is well matched to the strain magnitude, electrophysiological studies are needed to determine the encoding approaches. In addition to providing real-time wing state estimation, wing sensors could also help train the forward model and inverse model necessary for predictive motor control. These internal models are required for highly precise and fast aerial behaviours such as target interception (Mischiati et al., 2015) (Lin and Leonardo, 2017).

### Summary and conclusion

The wing sensory system is fascinating because it is directly implemented on the flight apparatus and mediates the control of flight. This work bridges our understanding of the wings biomechanics and the neurophysiology of wing sensors by providing the most complete characterization of wing sensor types, distribution, and wiring. In addition to guiding neurophysiological studies, our data enable future fluid-structure interaction simulations to illuminate how strain and flow sensing inform the spatial and temporal aerodynamic state of insect wings.

The amount of wing deformation scales with wing loading and wing size. Large insects (e.g. dragonflies) experience more wing deformation than small insects (e.g. fruit flies). It is important to recognize how flight control strategies must also change with the level of wing deformation control. In soft robotics terms, insect wings are underactuated (degrees of freedom > actuated degrees of freedom) yet extensively sensorized structures. Their venation patterns must not only produce predictable passive mechanical behaviors but also provide an appropriate substrate to support the number, density, and appropriate positioning of wing sensors to observe the relevant biomechanical events during flight. This integration of mechanical behavior and sensing for state estimation (sometimes termed “morphological computation”) makes insect wings an intriguing and tractable model for investigating the co-evolution of form, function, and the nervous system.

### Limitations of the study

To our knowledge, our morphological model is the most detailed whole insect wing used in this way to date. However, both the geometric model and the simulation have simplifications. The Young’s modulus of the material is uniform over the entire wing with no elastic joints (e.g. the nodus). The vein cross-sections are circular and filled whereas, in reality, they are often hollow and elliptical. We subjected the wing to a simple sinusoidal oscillation around a single axis (whereas rotation about three axes occurs in real flapping flight) and the motion is not influenced by fluid dynamics. The wing’s structural behaviour when interacting with aerodynamic forces is the subject of future work. Nevertheless, this current model illustrates the general strain propagation patterns that are necessary to infer how aeroelastic wing state might be encoded by the wing-mounted campaniform sensilla.

Our morphological and neuroanatomy data are complete, but the sensor identification has been based on comparison to similar systems in the published literature. To developed cell-type classification, some molecular work such as transcriptomics should be done in the future.

This work identifies the wing sensory location and starts to reconstruct the mechanical stimulus propagation on the wing blade. However, we cannot decode the sensory signals without a careful electrophysiological characterization of the sensor array. Our on-going work aims to correlate sensor responses to empirically measured wing deformation to reveal key features captured in the neural signals.

## STAR★Methods

### Key resources table

**Table:** 

REAGENT or RESOURCE	SOURCE	IDENTIFIER
**Chemicals, peptides, and recombinant proteins**		

Neurobiotin	Vector Labs	Cat# SP-1120; RRID: AB_2336606
DyLight 594-NeutrAvidin	Thermo Scientific	Cat #22842

**Deposited data**		

Anatomical data	This Paper	Dryad: https://doi.org/10.5061/dryad.h18931zn

**Software and algorithms**		

Fiji	Schindelin et al., 2012	https://imagej.net/software/fiji/
Matlab 2020a	Mathworks	https://au.mathworks.com/products/get-matlab.html?s_tid=gn_getml
Icy	de Chaumont et al. (2012)	https://icy.bioimageanalysis.org/

RStudio	RStudio	https://www.rstudio.com/products/rstudio/
NRecon 1.7.4.2	Bruker	https://www.bruker.com/
Dragonfly v3.6	Object Research Systems	https://www.theobjects.com/dragonfly/index.html
Rhinoceros 6	Robert McNeel & Associates	https://www.rhino3d.com/
AutoDesk Inventor Professional 2020	AutoDesk Inc.	https://www.autodesk.com/
ANSYS 2019 R3	ANSYS, Inc.	https://www.ansys.com/
ParaView 5.9.0	Kitware, Inc.	https://www.paraview.org/

### Resource availability

#### Lead contact

Further information and requests for resources and reagents should be directed to and will be fulfilled by the lead contact, Dr Huai-Ti Lin (h.lin@imperial.ac.uk).

#### Materials availability

This study did not generate new unique reagents.

### Experimental model and subject details

#### Insect specimens

Adult odonata specimens were selected to account for past studies and to cover a wide wing size range. They were collected in northern Virginia, USA (∗), south-east England, UK (∗∗), or otherwise obtained from the collection at the Natural History Museum in London, UK. They belong to two suborders: first, Anisoptera, the dragonflies, of which there are 13 families; members of the nine most populous/largest were studied here: 1. Aeshnidae, *Anax junius* (Drury, 1773)∗, 2. Chlorogomphidae, *Chlorogompus atkinsonii* (Fraser, 1925), 3. Cordulegastridae, *Anotogaster seiboldii* (Selys, 1854), 4. Corduliidae, *Hemicordulia tau* (Selys, 1871), 5. Gomphidae, *Hagenius brevistylus* (Selys, 1854) and *Stylyrus plagiatus* (Selys, 1854)∗, 6. Macromiidae, *Macromia indica* (Frasner, 1924), 7. Libellulidae, *Pantala flavescens* (Fabricius, 1798)∗, *Perithemis tenera* (Say, 1840)∗and *Sympetrum striolatum* (Charpentier, 1840)∗∗, 8. Petaluridae, *Petalura gigantea* (Leach, 1815), 9. Synthemistidae, *Idionyx stevensi* (Fraser, 1924). Second, Zygoptera, the damselflies, of which there are 35 families. The damselflies used here were from the 1. Calopterigidae, *Calopteryx maculata* (Palisot de Beauvois, 1807)∗ and 2. Coenagrionidae, *Argia apicalis* (Say, 1840)∗ and *Ischnura verticalis* (Say, 1839)∗. We used males in all our analyses, except for *P. tenera*, for which we used both males and females. In this species, the male wings are ∼10% shorter and have ∼20% more cross-veins than female wings. However, since most wing sensors are located on the longitudinal veins, the sexual dimorphism is not included in the discussion.

### Method details

#### Sample preparation for confocal imaging

Neurobiotin diffuses at a rate of ∼1 cm/24 h in axons of the diameter found in the Odonata wing, and insect tissue deterioration begins at approximately 48 hr postmortem. Thus, we focused our efforts on one of the smallest Anisoptera, *P. tenera*, and an Isoptera, *A. apicalis*, with comparable wingspan.

Insects were anesthetized on ice and fixed right side up on Sylgard-filled Petri dishes using pins. The right pterothoracic pleural wall and the head were removed, anterior nerves (nerves 1C and 2C in (Simmons, 1977)) were isolated, placed in a drop (∼0.7 μl) of distilled water inside a petroleum jelly bowl, and cut. Water was wicked away, replaced with ∼0.5 μl of the tracer solution (2 % w/v neurobiotin in dH2O, Vector Labs, SP-1120) and the well was sealed closed with petroleum jelly. Insects were kept refrigerated in a humid chamber for 48 hours to allow diffusion of the tracer. The wings were removed by cutting along the basal hinge and fixed in 2% PFA in phosphate-buffered saline with 0.1% Triton X-100 (PBS-T) overnight at 4°C with mild agitation, washed with PBS-T and bleached in 20% peroxide in PBS-T for 48 hr to remove most of the pigment from the cuticle.

When wings from young adults (few hours post-eclosion) were used, oxygen from bleaching formed within the bilayer of cuticle making up the wing blade. This can be explained by the presence of metabolites between the unfused sheets of exoskeleton reacting to the peroxide. At the end of bleaching cycle (indicated by the cuticle’s pale golden hues) the wings became fully inflated by the oxygen. The wings were briefly rinsed in copious amount of water, and placed inside a vacuum desiccator in a PBS-T-filled Petri dish. The low vacuum caused further delamination of the cuticular bilayer. The gas escaped via the openings at the wing base or, occasionally raptured the trailing edge seam. The wings were cut chordwise in half, post-fixed in 2 % PFA for 3 h @ RT, washed and stained with DyLight 594-NeutrAvidin (1:250, Thermo Scientific #22842). The staining reagent now had free/unencumbered access to the neurobiotin-labelled neurons, in PBS with 3% normal goat serum, 1% triton X-100, 0.5% DMSO @ RT with agitation for 2 days. Following washing with PBS-T, the wings were cut near their base and mounted in Tris-buffered (50 mM, pH 8.0) 80% glycerol with 0.5 % DMSO between two coverslips using 350 μm spacers. The wing bases were dehydrated in glycerol (5-80 %), then ethanol (25%-100 %), cleared and mounted in methyl salicylate, following modified protocol from (Ott, 2008).

For treatments of old wing specimens, samples were bleached and re-hydrated using alkaline peroxide (25% H2O2, 0.2% KOH in water) for 24-48 h and mounted in Tris-buffered (50 mM, pH 8.0) 80% glycerol with 0.5 % DMSO between two 60 mm-long coverslips. The mounting followed the same procedure as fresh wings.

#### Imaging and sensor distribution/placement mapping

All samples were imaged on Zeiss 880 upright confocal microscope. Serial optical sections were obtained at 7 μm with a FLUAR 5x/0.25 NA, 2.5 μm with a Plan-Apochromat 10x/0.45 NA objective, at 1.5 μm with a LD-LCI 25x/0.8 NA objective or at 0.5 μm with a Plan-Apochromat 40x/0.8 NA objective. Cuticle autofluorescence and DyLight 594-NeutrAvidin were imaged using 488 and 594 nm laser lines, respectively. The volumes obtained with the FLUAR 5x objective and tiling the wings were stitched in Fiji (http://fiji.sc/), processed in Icy (http://icy.bioimageanalysis.org/) and Photoshop (Adobe Systems Inc.).

The costa, radius and subcosta veins were scanned dorsally and ventrally with Plan-Apochromat 10x/0.45 NA objective (field of view 1.2 x 1.2 mm). To minimize collection time while maintaining high signal to noise ratio, the green autofluorescence of cuticle was excited with 405 and 488 nm lasers at maximum power and the images were collected using single-swipe and minimum pixel dwell time. The vertical aspect of each volume was adjusted to accommodate the imaged vein while the horizontal was kept constant at 1500 pixels. Maximum intensity projections were stitched manually in Photoshop and annotated in Adobe Illustrator (Adobe Systems Inc.) while referring to the volumes viewed in Fiji . 31 sensors were psuedorandomly selected on the forewing and hindwing of one sample for axon path length measurements. Sensors selection was to ensure measurements were taken from a wide variety of wing veins and locations, to avoid wing margin sensors dominating our dataset. Axon path lengths were measured in Fiji, and the minimal path length was determined using the guess and check approach. We constrained our analysis to only allow alternative axon paths that pass through innervated veins.

#### Assessment of backfill efficacy

To assess the completeness of neurobiotin labelling, we focused on the hind wing trailing edge bristles, as they are innervated by the longest axons (most challenging) and hence good indicators of labelling efficacy. We found that within 4.4mm stretch the sensory neurons innervating the bristles of the proximal half of trailing edge (i.e., axons passing through anal and cubitus posterior veins along with their tributaries) were all labelled (Figure S1D, top). However, within the distal 5.8 mm stretch of the trailing edge (i.e. axons traveling through media, radius veins and associated cross-veins), 2/23 dorsal and 4/33 ventral sensory neurons were not labelled (89.3 % efficacy) (Figure S1D, bottom). Projecting from the full labelling of the proximal half, we accounted for this ∼10% dropout at the distal portion.

#### Wing geometry modelling for structural analyses

A male *Sympetrum striolatum* dragonfly was euthanised in a freezer overnight. The entire body was stained by elemental iodine, which also had a desiccating effect (Boyde et al., 2014). The x-ray microtomography (μCT) scanning on the left hindwing was performed using a SkyScan 1172 scanner (Bruker, Belgium). The wing was mounted with the long axis vertical, aligned to the rotational axis of the scanner, and five sub-scans were performed. In total, 12445 images were taken at pixel size of 2.83 μm and exposure time of 2.6 s, with a rotation step of 0.08° for 180° rotation for each sub-scan. The source voltage and current were 65 kV and 153 μA, respectively. The raw images were processed in Bruker NRecon to obtain cross-sectional slices. The images were then imported to ORS Dragonfly v3.6 (Object Research Systems, Canada) for registration and three-dimensional reconstruction. A mesh file (PLY) was extracted by choosing an appropriate intensity threshold. After cleaning and reduction in MeshLab, the mesh was imported to Rhinoceros 6 (Robert McNeel & Associates, USA). The vein network was approximated with circular cross-section pipes whose diameter and joint positions were informed from the mesh. The membrane was generated for each cell surrounded by veins with uniform thickness of 10 μm, which was determined by sampling the measured cross section using the Dragonfly software. The veins and membranes were imported into AutoDesk Inventor Professional 2020 (Autodesk, Inc., USA) to merge them into a single body. The wing model was exported in STEP format and imported into ANSYS Mechanical Application 2019 R3 (ANSYS, Inc., USA), where the mesh for finite element analysis was generated for transient structural simulations.

#### Wing dynamic loading computational solid dynamics (CSD)

The transient structural simulation was performed with ANSYS Mechanical Application 2019 R3. The wing model mesh consists of quadratic tetrahedron elements. The uniform material property was assumed for simplicity, where density was 1200 kg m-3, Poisson ratio was 0.3, and the Young’s modulus was 5 GPa. These values were derived from previous work [S6], where the Young’s moduli were 6 GPa for vein and 3.75 GPa for membrane. Single-axis rotation around the wingbase resembling flapping is the simplest and most natural dynamic loading stimulus. Thus only 1 degree of freedom for rotation around the flapping axis (Figures 5A–5D) was prescribed, and other 5 (3 translational and 2 rotational) degrees of freedom were fixed. The flapping motion is described as:

$$\varphi =\varphi_{0}\;\sin\left(2\pi tf\right)\left(1-e^{-\gamma t}\right)\;$$

where φ is positional angle given at the wingbase in degrees (Figure S3D, blue line), φ_0_ is the desired wingbeat semi-amplitude and set to 30°, *t* is time in seconds, *f* is wingbeat frequency and set to 40 Hz, and *γ* is a factor to change how fast the amplitude approaches to φ_0_. The wingbeat amplitude increases gradually in this formulation so we can avoid the abrupt start and it is expected to arrive at the periodic deformation state earlier than a simple sinusoidal flapping. The factor *γ* as set to 30, which results in the wingbeat amplitude is 30% of φ_0_ for the 1st cycle but 67%, 84%, and 93% for 2nd, 3rd, and 4th cycles, respectively. At 10th cycle the amplitude is 99.9% of φ_0_. The wing started flapping from the mid-downstroke phase at time *t* = 0, and the computation was performed until time *t* = 16*T*= 0.4 s, where *T* = 1/*f* was the wingbeat period (i.e. 0.025 s). The time step size (time increment, *Δt*) for each computational step was determined to be 3×10^-4^ s after several preliminary trials. No external forces such as gravitational force or aerodynamic force were considered in this analysis.

The positional angle was defined such that the original attitude at mid-downstroke is 0, and negative angle at supination and positive angle at pronation. The wingtip path and wing positional angle at the wingtip were calculated for troubleshooting.

#### Mesh convergence check

Coarse and fine meshes were generated for mesh convergence verification, where coarse mesh has 845,039 nodes while the fine mesh has 1,226,124 nodes (Figures S3A and S3B). They showed slightly different low-frequency oscillation patterns in the wingtip positional angles (Figures S3C–S3H). For fair comparison, we took the wingbeat cycles where the mean positional angle during downstroke is the smallest for each case (25th cycle for coarse mesh and 30th cycle for fine mesh, Figures S3G and S3H). The spanwise strains in both meshes showed good agreement (Figure S3I). Thus we can confirm simulation convergence and show the results from fine mesh alone.

#### Strain analysis

The resultant strain field was evaluated in two ways: the normal elastic strain along two major longitudinal veins (ventral side of subcosta and dorsal side of radius), and the normal elastic strain contours on the wing surface at four time instants (pronation, mid-downstroke, supination, and mid-upstroke). Here, the normal elastic strain was computed for the wing radial direction for each instant by coordinate transformation using the instantaneous positional angle. We will refer this strain component as “spanwise strain” for short.

The period of low-frequency oscillation for the fine mesh case was around 8-10 wingbeat cycles (Figures S3D, S3F, and S3H). The strain along two veins for one low-frequency period (from the 22nd to 30th wingbeat cycles) showed some variation but the general trend was consistent, where the mean spanwise strain for the ventral subcosta and the dorsal radius show opposing temporal patterns (Figures S3J, S3K, and S3L). Therefore, in the main text, we show the representative results from the 28th wingbeat cycle starting from pronation (i.e., 27.75≤t/T≤28.75). For simplicity, each wingbeat cycle was described with $\hat{t}$ such that 0≤$\hat{t}$≤1, where $\hat{t}$ = 0 and 1 are pronation and $\hat{t}$=0.5 is supination. The spanwise strain along two veins were analysed in three ways. The raw strain along each vein for each instant was plotted (Figures 5E and 5F); the raw strain was normalised with the mean along the vein at the corresponding time (Figures 5G and 5H); and the mean strain was plotted against time. This way, we expected to see if the time-varying strain distribution can be decomposed into spatial and temporal components.

The spanwise strain contours on the surface were calculated in ANSYS CFD-Post 2019 R3. The mesh and strain for each time instance was exported in a Generic format using Command Editor with simple for-loop. The files and converted into VTK (legacy) format with a custom-written Python script and imported into ParaView 5.9.0 (KitWare, Inc., USA) for visualisation.

### Quantification and statistical analysis

#### Cell body volume and axon diameter quantification

For the cell body and axon diameter quantification, equally distributed wing sensors were selected from the right hindwing of one *P. tenera* sample. Cell body volumes were quantified by first manually identifying the intensity threshold in ITK SNAP (http://www.itksnap.org/) which was used to segment the cell body volume voxels from the background image and axon. Second, the identified threshold of 1800 was implemented in a custom written Fiji macro to automatically segment all images and obtain the resulting cell body volume. The results were validated with the manually obtained volumes from ITK SNAP for randomised samples. Axon diameter was measured at 20 μm proximally from each cell body in Fiji.

Assuming a cylindrical axon with homogenous membrane properties, the neural conduction speed *v* can be estimated as (Tasaki, 2004):

$$v=\;\sqrt{\frac{d}{8\cdot \rho \cdot C^{2}\cdot R}}$$

where *d* is the axon diameter, *ρ* is the resistivity of the axoplasm, *C* is the membrane capacitance per unit area, and *R* is the resistance per unit area. The conduction latency *t*, therefore, can be represented as:

$$t=\frac{L}{v}\;\propto \;\frac{L}{\sqrt{d}}\;d=a{\left(L_{w}+b\right)}^{2}$$

where *L*_w_ is the axon length in the wing, a is a scaling factor and b is the axon length offset accounting for the distance from the wingbase to the thoracic gangalion. We performed nonlinear least squares fit to this relationship with data from the two sensor types examined. We also performed linear regression to provide a referenc comparison.

#### Wing sensor distribution quantifications

Based on the annotations, sensors were counted and their positions digitized in Fiji. The sensor density was defined as the inverse of the averaged sensor spacing over two consecutive sensors (sensors/mm). The sensor position was normalised by the spanwise wing length for comparison across species. Sensor density was smoothed by a moving average over 7 data points and subsequently normalised by the mean sensor density of the respective sensor type on the vein to facilitate relative comparisons across species.

The relationship between sensor count and wing length for each sensor type was analysed via an ordinary least squares (OLS) regression in Rstudio (Rstudio Team, 2020). The lower and upper confidence intervals (2.5% and 97.5%) of the OLS regression were calculated. In this analysis, the sensor count for each sensor type corresponded to the sum of the sensors of the first three anterior veins (costa, subcosta and radius).

#### Wing axon path distance measurement

31 sensors on a forewing and hindwing were pseudorandomly selected, to achieve an even sampling of sensors across the wing areas and avoid highly sensorised veins (ie the costa) from skewing our measurements. The length of the axon path was measured from confocal imagery in Fiji. Alternative axon paths were determined manually, and the shortest identified path was determined in Fiji by using the guess-and-check approach. In this analysis we constrained our alternative paths to only allow passage through innervated veins.

Wing innervation and sensor distribution of an eastern amberwing dragonfly, *Perithemis tenera*. Labelled neurons in the whole mount preparation of the wing imaged with a 5x objective are shown in green. Placement of five classes of wing sensors are shown as examples (teardrop markers): campaniform sensilla, wing margin bristle-bump complexes, bristle-bump complexes, isolated bristles and multipolar receptors. Representative sensors are shown in three-dimensional rotating views, generated from high-magnification confocal data.

Visualization of the spanwise strain for a common darter dragonfly, *Sympetrum striolatum* in the flapping-wing structural dynamics simulation. Spanwise strain surface contours in the wing coordinates (left) and spanwise strains on two major longitudinal veins (right). See Methods for the definitions. The wingbeat cycles shown are 21.75 ≤ t/T ≤ 30.75, starting from pronation.

## Data Availability

• Raw microscope images, sensor measurement values, and CFD model information have been deposited at Dryad and are publicly available as of the date of publication. DOIs are listed in the key resources table. • This paper does not report original code. • Any additional information required to reanalyze the data reported in this paper is available from the lead contact upon request.
