# Supplementary material for: Systematic characterization of wing mechanosensors that monitor airflow and wing deformations
Source: iScience. 2022 Mar 22;25(4):104150. doi: 10.1016/j.isci.2022.104150 (PMC9018384; doi:10.1016/j.isci.2022.104150)
Supplement: Supplementary file 3 — Document S1. Figures S1–S3 and Table S1 [file mmc1.pdf]

iScience, Volume *25*

Supplemental information

Systematic characterization of wing mechanosensors that monitor airflow and wing deformations

Joseph Fabian, Igor Siwanowicz, Myriam Uhrhan, Masateru Maeda, Richard J. Bomphrey, and Huai-Ti Lin

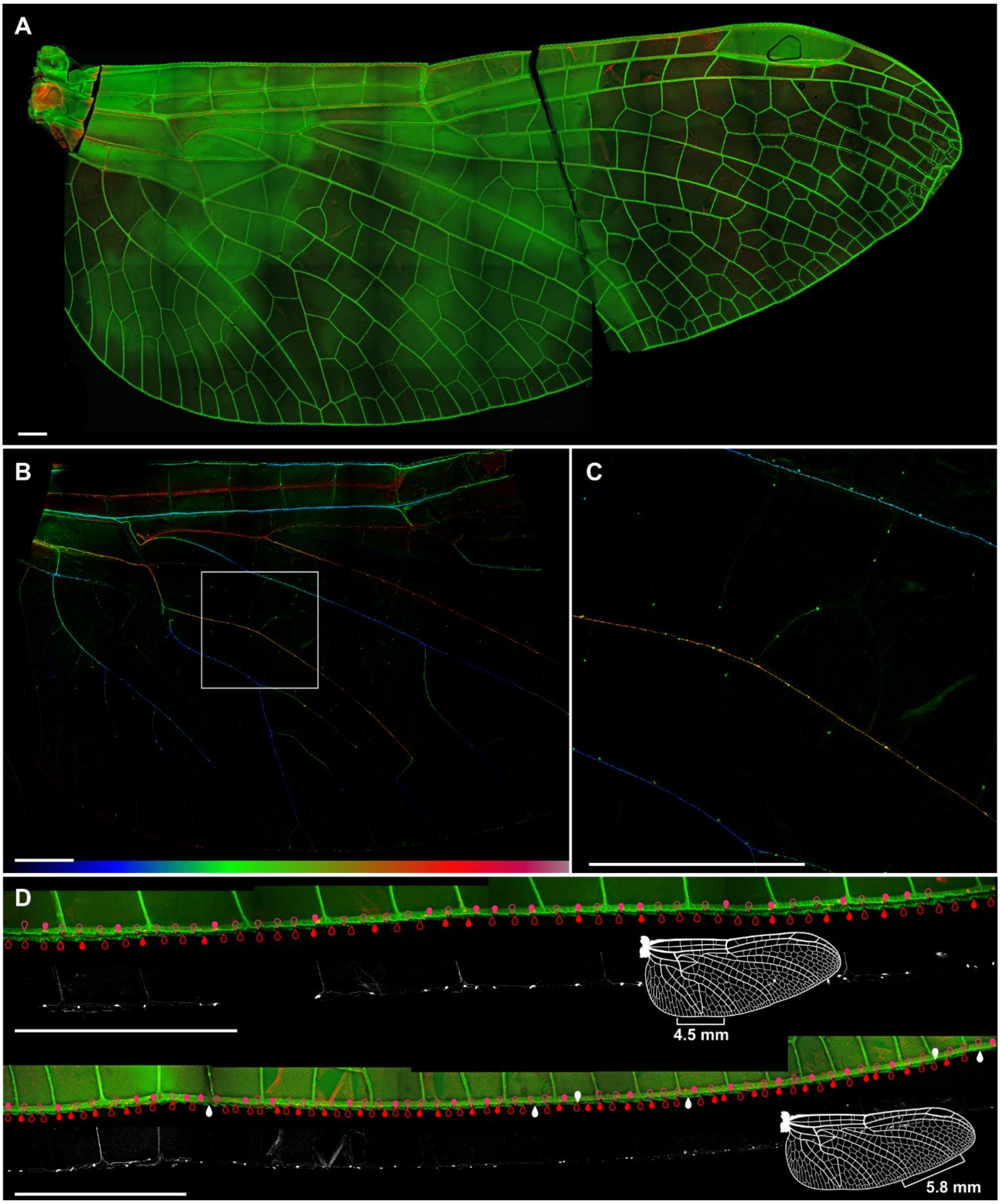

Figure. S1. Examples of confocal imagery following fluorescent labelling of wing sensory neurons. Related to Figure 1.

(A) *Perithemis tenera* hind wing; maximum intensity projection of stitched volumes collected with the FLUAR 5x objective. Cuticle autofluorescence – green; DyLight 594-neutravidin – red. **(B)** Depth color-coded projection of the red channel. Soma of various sensory neurons appear as dots. **(C)** Area indicated in **(A)** by the box. **(D)** Assessment of the efficiency of the backfill using *P. tenera* hind wing trailing edge bristle-bump complexes as a proxy. Filled markers indicate filled sensory neurons.

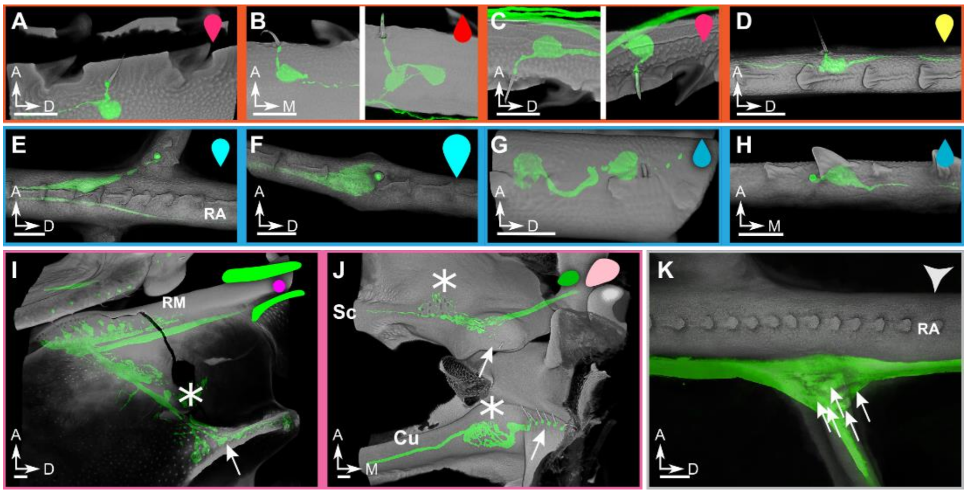

Figure. S2. Examples of wing veins and base sensors of the damselfly *Argia apicalis*. Related to Figure 3.

Most structures are analogous to the dragonfly. **(A-D)** air flow sensors; **(A)** dorsal costa bristle-bump complex; **(B)** ventral costa bristle-bump complex; left: single-innervated; right: double-innervated bristle. **(C)** trailing edge bristle-bump complex; left: single-innervated; right: double-innervated bristle. **(D)** short isolated bristle. **(E-H):** strain sensors; (**E**) Campaniform sensilla (CS) of a cross vein medial to pterostigma; (**F**) large dorsal radius anterior CS immediately distal to pterostigma; **(G)** ventral media CS proximal to the wing base; (**H**) one of ventral media posterior CS. **(I-K)** wing base sensors. **(I)** crevice organ (i.e. campaniform sensilla) – two parallel fields of directionally tuned/elongated CS dorsally at the base of radius/media (RM) vein. Asterisk marks the dorsal insertion site of a wing base chordotonal organ. Arrow – CS field of axillary complex posterior ridge. **(J)** Ventral view of the subcostal and cubitus vein base. Arrows point to hair plates, asterisks mark CS fields. **(K)** Multipolar receptor at the junction of radius and a cross vein posterior to the nodus. Arrows point to several large cell bodies. All the images show mechanosensors of the right forewing. Cuticle's autofluorescence shown in grey, neurons labelled with Neurobiotin/DyLight-594-Neutravidin – in green. Scale bars: 25 µm. The symbols in the upper right corner of each panel correspond to the markers showing placement of sensors in Figure 3.

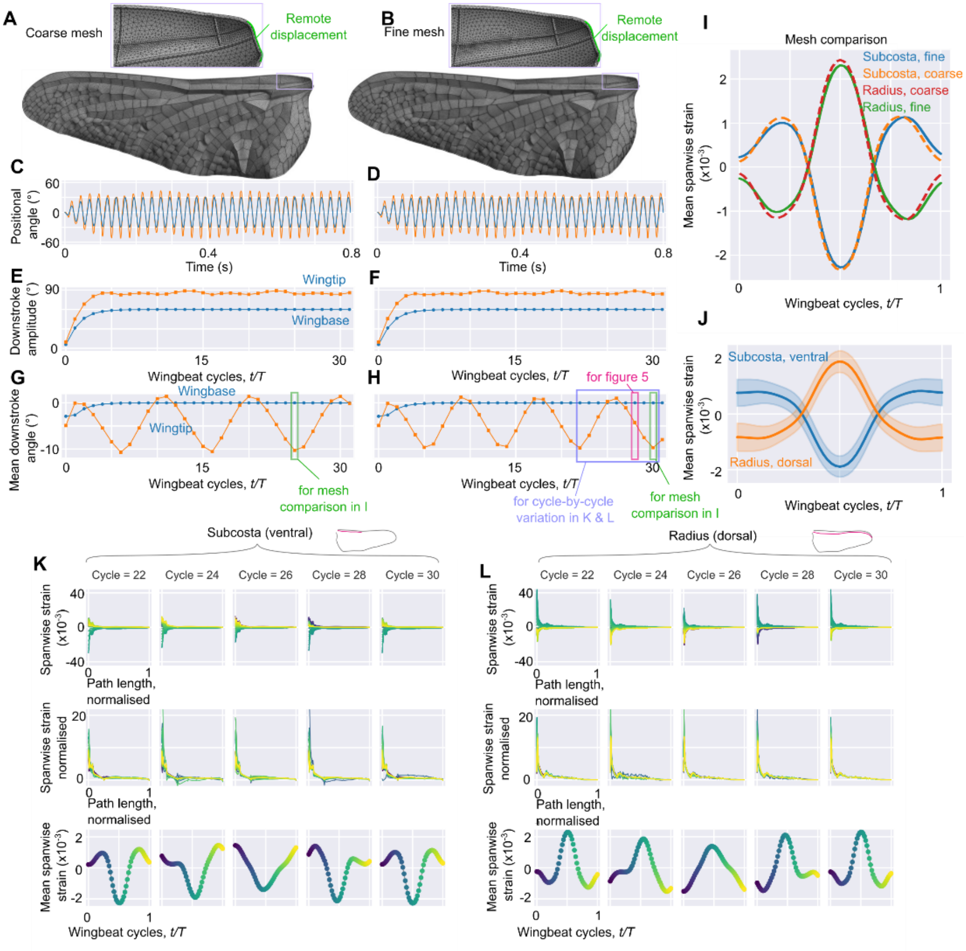

Figure. S3. Mesh convergence and cycle-by-cycle variation in spanwise strain. Related to Figure 8.

Coarse **(A)** and fine **(B)** meshes. Enlarged views show the mesh near the wing base. The green region is the faces that the “remote displacement” boundary condition was applied in ANSYS Mechanical to drive the wings. Positional angles **(C,D)**, wingbeat amplitude for downstroke **(E,F)**, and mean positional angle for downstroke **(G,H)**, where blue lines are for wingbase and orange lines are for wingtip. Note the 0 mean positional angle means the wing flaps equal amount for dorsal and ventral directions. In the current case, the mean positional angle tends to be negative, indicating the downward bending. The mean (along each of 2 vein) spanwise strains for coarse and fine meshes **(I)**, where blue and green solid lines are subcosta and radius in fine mesh, respectively, and orange and red dashed lines are subcosta and radius in coarse mesh, respectively. The ensemble average for the mean spanwise strain over nine consecutive wingbeats ($21.75 \leq t/T \leq 30.75$) (J), where blue line is subcosta and orange line is radius. Mean and ±1 SD are shown. The cycle-by-cycle variation in spanwise strain for subcosta **(K)** and radius **(L)**. Cycle 22 corresponds to $21.75 \leq t/T \leq 22.75$, and so on.

	Mean sensor density (1/mm)												Wing length (mm)	Sensor count
	C(d)		Sc(d)		R(d)		C(v)		Sc(v)		R(v)			
**Species**	**cs**	**bb**	**is**	**cs**	**bb**	**sis**	**cs**	**bb**	**cs**	**bb**	**sis**	**is**		
I.verticalis		6.69						9.64					1.15	202
P.tenera		7.81	5.58	2.2	2.92			11.98	10.73			3.71	2.07	519
A.apicalis		2.95						5.94					2.46	168
S.striolatum	1.45	4.99	3.78	1.06	2.65			7.83		2.19		3.45	2.95	547
C.maculata		4.93				6.71	2.27	8.86					3.06	392
I.stevensi	1.94	10.63	3.75	1.33	2.48			15.57	6.71	3.88		11.19	3.31	1080
H.tau	2.38	5.5	7.24	1.45	2.7			9.12	4.46	2.39		12.16	3.63	734
S.plagiatus	3.08	4.01		5.52	1.5		2.10	6.21	7.18	1.45			3.74	600
C.atkinsoni	2.55	5.18		6.96	2.48		4.25	8.74	6.69			12.64	4.05	900
P.flavescens	1.16	4.94	12.37	1.06	2.37			7.73		2.63		14.41	4.33	844
M.indica	1.11	6.72		2.86	3.27			15.18	3.37	3.05		7.3	4.76	1420
A.junius		5.47	2.99	2.35	2.24		2.83	6.47	5.08	2.2		7.55	5.36	1065
P.gigantea	2.98	4.22		5.36	2.00		3.58	4.02	7.38	1.43	0.98	4.45	5.41	793
A.sieboldii	2.34	3.93	1.64	5.49	1.99	3.73	1.75	7.26	6.99		1.64		5.44	958
H.brevistylus	1.77	3.93	2.03	3.98	1.81	2.62	2.63	6.55	5.81		2.03		5.45	766

Table S1. Mean sensor density, wing length and sensor count of 15 most studied Odonata species. Related to Figure 7.

Mean sensor density and sensor counts are presented for the three major longitudinal veins Costa (C), Subcosta (Sc) and Radius (R) on dorsal (d) and ventral (v) wing side. Mean sensor density is presented for campaniform sensilla (cs), isolated bristles (is), bristle-bump complexes (bb) and small bristles in the range of a bump (sis). The last sensor type was assigned to the bristle-bump complexes in the article because the bristles had the same morphology and could be associated to a nearby cuticular bump.
